# Development and Validation of the Dementia‐Perceived Abuse and Neglect Questionnaire (D‐PANQ) among Family Caregivers of People Living with Dementia

**DOI:** 10.1002/alz.094768

**Published:** 2025-01-09

**Authors:** Seelia Peter, Elsa Sanatombi Devi, Mukhyaprana Prabhu, Rajeshkrishna Bhandary, Jyothi Chakrabarthy, Binil V

**Affiliations:** ^1^ Manipal College of Nursing, Manipal Academy of Higher Education, Manipal, Karnataka India; ^2^ Manipal College of Nursing, Manipal Academy of Higher Education, Manipal, Karnataka, India, Manipal, Karnataka India; ^3^ Kasturba Medical College, Manipal Academy of Higher Education, Manipal, Manipal, Karnataka India

## Abstract

**Background:**

Abuse of older adults is a sociopolitical issue that is often hidden. People living with dementia are more vulnerable to abuse due to their cognitive and physical impairments. Caring for a person with dementia is quite challenging. The caregivers abuse the older adults they take care of due to the increasing caregiving demands, caregiver burden, stress, depression, or anxiety. The caregivers often know what the potentially abusive behaviors are and what not to do, but at times they tend to behave in an abusive way. Some of the situations that happen in a day that are abusive are not perceived as such by them due to the normalization of what they have been doing for years. A vignette‐based tool helps to collect data regarding sensitive issues like abuse. The objective of this study was (1) to analyze the psychometric properties of the Dementia‐Perceived Abuse and Neglect Questionnaire (D‐PANQ) among family caregivers of people living with dementia regarding abuse using a newly developed vignette‐based tool. (2) to assess the perception of family caregivers of people living with dementia regarding abuse.

**Method:**

This cross sectional study will be completed among 300 caregivers of people living with dementia. The study is ongoing, with data collected from 190 caregivers. The tool is a 22‐item, 4‐point scale with a maximum score of 66. The higher scores show a high perception of abuse, and the low scores show a low perception of abuse.

**Result:**

The interim analysis among 190 caregivers shows that the perception of abuse is high. The mean DPANQ score was 42.86, with a standard deviation of 16.01. The reliability of the tool was established by Cronbach’s alpha. It was found to be 0.82. Further analysis of the questionnaire’s feasibility, reliability, internal consistency, content validity, and construct validity (factor analysis and convergent validity) will be done once the study is completed.

**Conclusion:**

The initial analysis shows that the tool is valid, reliable, and feasible to assess the perception of family caregivers of people living with dementia regarding abuse, and this would be the first tool to assess the perception of dementia carers towards abuse and neglect.

